# Characterization of *Pediococcus ethanolidurans* CUPV141: A β-D-glucan- and Heteropolysaccharide-Producing Bacterium

**DOI:** 10.3389/fmicb.2018.02041

**Published:** 2018-09-04

**Authors:** María G. Llamas-Arriba, Adrián Pérez-Ramos, Ana I. Puertas, Paloma López, María T. Dueñas, Alicia Prieto

**Affiliations:** ^1^Chemistry Faculty, Department of Applied Chemistry, University of the Basque Country (UPV/EHU), San Sebastián, Spain; ^2^Department of Microorganisms and Plant Technology, Biological Research Center, Spanish National Research Council (CIB-CSIC), Madrid, Spain

**Keywords:** *Pediococcus ethanolidurans*, β-glucan, *gtf*, heteropolysaccharides, priming-glycosyltransferase, plasmid, adhesion

## Abstract

*Pediococcus ethanolidurans* CUPV141 is an exopolysaccharide (EPS)-producing lactic acid bacterium, first isolated from Basque Country cider (Spain). Physicochemical analysis of the EPS synthesized by the bacterium revealed that CUPV141 produces mostly a homopolysaccharide (HoPS), characterized as a 2-substituted (1,3)-β-D-glucan, together with a small quantity of a heteropolysaccharide (HePS) composed of glucose, galactose, glucosamine, and glycerol-3-phosphate, this being the first *Pediococcus* strain described to produce this kind of polymer. On the contrary, an isogenic strain CUPV141NR, generated by chemical mutagenesis of CUPV141, produced the HePS as the main extracellular polysaccharide and a barely detectable amount of 2-substituted (1,3)-β-D-glucan. This HoPS is synthesized by the transmembrane GTF glycosyltransferase (GTF), encoded by the *gtf* gene, which has been previously reported to be located in the pPP2 plasmid of the *Pediococcus parvulus* 2.6 strain. Southern blot hybridization revealed that in CUPV141 the *gtf* gene is located in a plasmid designated as pPE3, whose molecular mass (34.4 kbp) is different from that of pPP2 (24.5 kbp). Analysis of the influence of the EPS on the ability of the producing bacteria to adhere to the eukaryotic Caco-2 cells revealed higher affinity for the human enterocytes of CUPV141NR compared to that of CUPV141. This result indicates that, in contrast to the 2.6 strain, the presence of the HoPS does not potentiate the binding ability of *P. ethanolidurans*. Moreover, it supports that the phosphate-containing bacterial HePS improved the interaction between *P. ethanolidurans* and the eukaryotic cells.

## Introduction

Some lactic acid bacteria (LAB) produce exopolysaccharides (EPS), extracellular polymers that may remain tightly attached to the bacteria, constituting a capsule, or may be released to the environment (De Vuyst et al., 2001). These polymers often possess useful properties, such as improvement of the rheological properties of food and even beneficial effects for health as prebiotics and immunomodulators (Bajpai et al., 2016; Caggianiello et al., 2016). The EPS can be homopolysaccharides (HoPS) or heteropolysaccharides (HePS), constituted, respectively, by one or various types of monosaccharides (Werning et al., 2012). Only one protein is responsible for the synthesis and the extracellular location of the HoPS, whereas the HePS synthesis and secretion requires the joint action of several proteins usually encoded by genes located in operons (Sanlibaba and Aybige Çakmak, 2016). A priming-glycosyltransferase (priming-GTF or p-GTF), which is phosphorylated by a tyrosine kinase (Minic et al., 2007), catalyzes the first step of the synthesis of HePS by the formation of a phosphoanhydride bond between the first hexose-1-phosphate of the repeating unit and an undecaprenyl phosphate lipid carrier anchored to the membrane. Then, the repeating-unit is synthesized, by sequential transfer of nucleotide sugar residues onto the growing chain by other glycosyltransferases (Whitfield, 2006). HePS are mainly composed of different ratios of glucose, galactose and rhamnose, and occasionally, they can be substituted by amino-sugars or polyols (glycerol) as well as by glucuronic acids and also phosphates (Badel et al., 2011). For LAB and bifidobacteria, genetic studies and characterization of the EPS produced by them have shown that these bacteria are able to synthesize more than one EPS. Characterization of these polymers has revealed that some strains produce HoPS and HePS (Ibarburu et al., 2007, 2015; Puertas et al., 2018) or several HePS (Remus et al., 2012; Salazar et al., 2012; Hidalgo-Cantabrana et al., 2013; Lee et al., 2016). In addition, HePS-producing *Streptococcus thermophilus* strains have been widely used for fermentations to generate dairy products as cheeses and yogurts because of the rheological properties their EPS confer to the final products (De Vuyst et al., 2003; Ravyts et al., 2011; Wu et al., 2011; Kanamarlapudi and Muddada, 2017). However, until now simultaneous production of HePS and HoPS by a pediococcal strain has not been reported. The most common EPS produced by strains belonging to this genus is a 2-substituted (1,3)-β-D-glucan (β-D-glucan) (Llaubères et al., 1990; Dueñas-Chasco et al., 1997) synthesized by the GTF glycosyltransferase (GTF) (Werning et al., 2008, 2014). This HoPS is produced by LAB isolated from alcoholic beverages: *Pediococcus damnosus* or *Oenococcus oeni* strains in wines (Lonvaud-Funel and Joyeux, 1988; Dols-Lafargue et al., 2008), *Lactobacillus* and *Pediococcus* strains in ciders (Dueñas et al., 1995; Fernández et al., 1995; Ibarburu et al., 2007; Garai-Ibabe et al., 2010a; Puertas et al., 2018) and *Lactobacillus* strains in beers (Fraunhofer et al., 2018). The GTF is encoded by the *gtf* gene, which is generally present in plasmids (Lonvaud-Funel et al., 1993; Werning et al., 2006), although a chromosomal location has been observed in *O. oeni* (Werning et al., 2006; Dols-Lafargue et al., 2008).

The interest in the identification and characterization of new LAB producing the β-D-glucan is due to the fact that this polymer immunomodulates human macrophages, and its presence increases the adhesion capability to enterocytes of the producing bacteria (Fernández de Palencia et al., 2009; Garai-Ibabe et al., 2010b). Moreover, this EPS has prebiotic activity (Russo et al., 2012; Pérez-Ramos et al., 2016b) and confers resistance to both technological and gastrointestinal stresses to lactobacilli upon heterologous expression (Stack et al., 2010).

This work reports on the characterization of *Pediococcus ethanolidurans* CUPV141, a novel strain isolated from cider that produces the 2-substituted (1,3)-β-D-glucan and a phosphorylated HePS. As far as we know this is the first instance of detection of this species in cider and the first characterized example of a *Pediococcus* producing HoPS and HePS. Moreover, the presence of these two EPS seems to play a role for adhesion of the bacteria to biotic surfaces.

## Materials and Methods

### Bacterial Strains and Growth Conditions

The EPS-producing *P. ethanolidurans* CUPV141 strain was isolated from Basque Country (Spain) ropy cider (containing 6% ethanol and at pH 3.4–3.8) as previously described (Dueñas et al., 1995) in Carr-agar medium (g/L; yeast extract, 4; casaminoacids, 5; DL malic acid, 5; glucose, 20; KH_2_PO_4_, 0.5; KCl, 0.425; CaCl_2_⋅2H_2_O, 0.125; MgSO_4_, 0.125; MnSO_4_, 0.0025) supplemented with pimaricin at 50 mg/mL to avoid the growth of yeasts and molds. The isogenic, non-ropy strain *P. ethanolidurans* CUPV141NR was generated in this work by chemical mutagenesis with the antibiotic novobiocin (Sigma-Aldrich) at a final concentration of 50 μg/mL. The *P. parvulus* 2.6 strain (Dueñas-Chasco et al., 1997) was used for comparative purposes. All strains were stored at -80°C in MRS medium (De Man et al., 1960) containing 20% glycerol. The experimental assays were performed in MRS medium pH 5.5 at 28°C and under an atmosphere containing 5% CO_2_. For EPS isolation, a semi-defined medium (SMD) was used (Dueñas-Chasco et al., 1997).

### Genomic and Plasmidic DNA Preparations

For genomic DNA extraction, NucleoSpin^®^ Tissue kit (Macherey-Nagel) was used according to the manufacturer’s instructions and supplementing the lysis buffer with lysozyme (Sigma-Aldrich) at 30 mg/mL and mutanolysin (Sigma-Aldrich) at 2 U/μL. Once isolated, samples were stored at -20°C until use.

Total plasmid DNA preparations of *P. ethanolidurans* CUPV141 and CUPV141NR strains were obtained and purified by isopycnic CsCl density gradient to eliminate non-supercoiled forms of the plasmids as previously described (Pérez-Ramos et al., 2017b). Plasmidic samples were maintained at -80°C until use.

Fluorescent quantification of the DNA in genomic and plasmidic DNA preparations was determined with a Qubit fluorometer using the Qubit HS dsDNA Assay Kit (Molecular Probes).

### 16S rDNA Amplification by PCR

The template for PCR amplification was genomic DNA from *P. ethanolidurans* CUPV141. The flanking primers 616V and 630R (Ehrmann et al., 2003) and the internal primer 699R (Arahal et al., 2008) were used to obtain two amplicons (1466 and 1009 pb) of the 16S rRNA gene. The PCR products were purified with a NucleoSpin^®^ Gel and PCR Clean-up kit (Macherey-Nagel) according to the manufacturer’s instructions. Subsequent sequencing reactions were performed at Secugen (Madrid, Spain). The DNA sequences obtained were used as templates for the identification of the strain in the public database EZBioCloud^1^ (Kim et al., 2012).

### Quantification of the 2-Substituted (1,3)-β-D-Glucan Produced by *P. ethanolidurans* Strains

A competition enzyme-linked immunosorbent assay (ELISA) was performed for the specific detection of the EPS synthesized by strains of *P. ethanolidurans*, based on *S. pneumoniae* serotype 37 antibody, as previously described (Werning et al., 2014). The EPS of *P. parvulus* 2.6 was isolated according to Notararigo et al. (2013) and immobilized in each well of a 96-Well Nunc-Immuno MicroWell MaxiSorp plate (Thermo Fisher Scientific). EPS quantification was performed using a standard curve of serial dilutions of the purified *P. parvulus* 2.6 EPS dissolved in PBS, generated by competition for the primary antibody.

To quantify the amount of EPS released to the growth medium or attached to the bacteria, *P. ethanolidurans* strains were grown in MRS medium (1 mL) to a final concentration of 1 × 10^8^ colony forming units (cfu)/mL in 1.5 mL Eppendorf tubes. Then, the cultures were centrifuged (9300 ×*g*, 4°C, 10 min), the supernatants were transferred to another Eppendorf tube and the bacteria were resuspended in 1 mL of phosphate buffered saline (PBS) pH 7.2. Dilutions of the culture supernatants and of the bacterial suspension in PBS were used for quantification, measuring the OD at 415 nm in a microtiter plate reader model 680 (Bio-Rad). The determinations were performed in triplicate.

### Characterization of the *gtf* and *p-gtf* Genes

Plasmid DNA from the *P. ethanolidurans* CUPV141 strain was used to determine the 1,704 bp nucleotide sequence of the *gtf* gene by the dideoxy method at Secugen as previously described for the *gtf* gene of *P. parvulus* strains (Garai-Ibabe et al., 2010b).

For detection of the priming-GTF coding gene (*p*-*gtf*), degenerate primers (Provencher et al., 2003) and genomic DNA from the two *P. ethanolidurans* strains were used for DNA amplification. The 20 μL reaction mixtures for each sample contained: 1 U of Taq DNA polymerase (Sigma), 1X PCR Buffer (Sigma), 2.5 mM MgCl_2_ (Sigma), 0.1 mM dNTP mixture (TaKaRa), 6.25 mM of each primer, and 200 ng of total template DNA. Conditions for the PCR were as follows. First, an incubation at 94°C for 9 min. Then, 5 cycles at 94°C for 30 s, 62°C for 31 s and 72°C for 32 s. Finally, 40 cycles at 94°C for 30 s, 52°C for 31 s, and 72°C for 32 s.

After fractionation in 2.5% agarose gels, the amplicons were purified using the ‘NucleoSpin^®^ Gel and PCR Clean-up’ kit, according to the manufacturer’s instructions, and the nucleotide sequence determined at Secugen.

### Informatics Analysis of Genes and Inferred Protein Sequences

The amino acid sequence of the GTF was inferred from the nucleotide sequence of the *gtf* gene with EditSeq 15 software (DNASTAR^®^ Lasergene 15). The sequences of the protein, the *gtf* gene as well as of the DNA fragment of *p-gtf* and its translated product were compared with those of other bacteria, deposited at the National Center for Biotechnology Information (NCBI) database^2^, using the Basic Local Alignment Search Tool (BLAST^3^). Multiple sequence alignments (MSA) of the sequences obtained in the search were performed with MegAling Pro 15 software (DNASTAR Lasergene 15) using the Clustal Omega algorithm. In addition, phylogenetic trees were obtained using Tamura-Nei (Tamura and Nei, 1993) or Kimura (Kimura, 1983) metrics for DNA and protein sequences, respectively. Finally, mutations in the amino acid sequence of the GTF of each bacterium were gathered in a secondary structure model of the *P. parvulus* 2.6 enzyme, previously inferred using the SOSUI program (Werning et al., 2006).

### Detection of the *gtf* Gene by Southern Blot Hybridization

Genetic localization of the *gtf* gene was performed following the protocol previously described (Pérez-Ramos et al., 2017b). Briefly, samples of plasmid DNA preparations from *Pediococcus* strains and from *Escherichia coli* V517 (Macrina et al., 1978) were fractionated by electrophoresis in a 0.7% agarose gel and DNA molecules were revealed by staining with ethidium bromide at 0.5 μg/mL. The gels’ images were obtained with Gel Doc 200 (Bio-Rad). Plasmids from *E. coli* V517 were used to generate a standard curve in which their relative migration in the gel was represented *versus* their known size (Macrina et al., 1978), which was used to determine the molecular mass of the pediococcal plasmids. The plasmidic DNA bands were transferred to a nylon membrane prior to hybridization. The internal regions of the *gtf* gene used as probes were generated by PCR amplification, in a reaction catalyzed by Phusion High Fidelity Polymerase (New England BioLabs), using as substrate total plasmid DNA preparation of *P. parvulus* 2.6 and the previously described primers GTFSF and GTFSR (Werning et al., 2006). Then, the amplicon was labeled with digoxigenin-dUTP using the DIG high prime DNA labeling and detection starter kit II (Roche). Each DIG-labeled DNA probe (25 ng/mL) was used for hybridization at 40°C following the specifications of the kit’s supplier. The hybridization bands were revealed with the chemiluminescent substrate CSPD, and the signals were detected with the LAS-3000 imaging system (Fujifilm).

### Agglutination Immunological Analysis

Agglutination tests were performed with *S. pneumoniae* type 37-specific antisera according to the protocol previously described (Werning et al., 2006). Briefly, cultures in late-exponential phase (1 × 10^9^ cfu/mL) from the two *Pediococcus* strains were centrifuged at 8609 ×*g* for 47 min at 4°C and after removal of the supernatants, the sedimented bacteria were concentrated 100-fold by resuspension in PBS pH 7.2 with vigorous vortexing. Then, 10 μL of each bacterial suspension was mixed with 1 μL of anti-type 37 antibody, and incubated for 2 h at 4°C. Afterwards, each sample (4 μL) was observed by phase contrast microscopy using a Leica DM 1000 microscope.

### Isolation of EPS

*Pediococcus ethanolidurans* CUPV141 and CUPV141NR were grown in MRS broth for 24 h. Then, the volume of bacteria corresponding to a 2% inoculum was sedimented by centrifugation (18500 ×*g*, 10 min, 4°C), resuspended in fresh MRS pH 5.5 medium and incubated at 28°C in a 5% CO_2_ atmosphere for 24 h. Finally, a 2% inoculum was sedimented again, in the same conditions, to inoculate the final fermentation in SMD pH 5.5 medium (Dueñas-Chasco et al., 1997). When the cultures reached the late-exponential phase of growth, bacteria were sedimented by centrifugation of the cultures (18500 ×*g*, 20 min, 4°C), and the EPS were precipitated from the supernatants by addition of 3 volumes of cold absolute ethanol and kept at -20°C for 16 h. Afterwards, the polymers were recovered by centrifugation (18500 ×*g*, 10 min, 4°C) and the crude EPS were washed three times with 80% (v/v) cold ethanol and dialyzed in 12–14 kDa MWCO membranes (Iberlabo) against distilled water, freeze-dried and kept at room temperature.

Lyophilized EPS were dissolved in ultrapure water (0.1 mg/mL) and concentration was estimated from the neutral carbohydrate content as determined by the phenol-sulphuric acid method (Dubois et al., 1956) using glucose as standard.

### Partial Characterization of the Crude EPS

*Pediococcus ethanolidurans* CUPV141 and CUPV141NR were incubated in MRS broth for 21 h. Then, the volume corresponding to an OD_600_ of 1.0 was centrifuged (9600 ×*g*, 10 min, 4°C). Supernatants were discarded and the sediments were resuspended in 0.5 mL of PBS pH 7.2. Then, in order to visualize similarities or differences in the EPS production between the two *P. ethanolidurans* strains, transmission electron microscopy (TEM) was used following the protocol of Zarour et al. (2017) with modifications. Briefly, a drop of each solution was independently deposited on a carbon film copper grid, previously hydrophylized by a glow discharge process of *ca.* 30 s, and the preparations were washed with water for 15 s. Then, each grid was stained for 15 s with a uranyl acetate water solution (0.5% w/v) in order to improve the image contrast, and washed again with water. Finally, the samples were air-dried and examined in a TECNAI G2 20 TWIN (FEI) microscope, operating at an accelerating voltage of 200 kV in a bright-field image mode, at the Microscopy Service of the University of Basque Country (UPV/EHU). Monosaccharide composition of the polymers, as well as methylation analysis for the elucidation of the *O*-glycosidic linkages involved in the structure of the EPS were developed following the protocols described by Notararigo et al. (2013). Finally, proton nuclear magnetic resonance (^1^H NMR) analysis of the EPS produced by the ropy strain was performed as previously described (Dueñas-Chasco et al., 1997) at the UPV/EHU NMR Service (SGIker).

### Adhesion Properties

#### Self-Aggregation Assay

*Pediococcus ethanolidurans* CUPV141 and CUPV141NR strains were grown in MRS medium pH 5.5 (1% inoculum) for 15 h. Then, the volume corresponding to 1 × 10^8^ cfu/mL was centrifuged (12000 ×*g*, 10 min, 4°C) and after removal of the supernatant, 1 mL of fresh MRS pH 5.5 medium was added. Two tubes of each strain were incubated at 28°C. Samples were recovered at 5 and 24 h (one tube of each strain for each time) as follows: the tubes were gently centrifuged (5000 ×*g*, 3 min, 4°C), and after removal of the supernatants the bacteria were carefully resuspended in 50 μL PBS pH 7.2. Aliquots of 5 μL of this suspension were visualized by phase contrast microscopy using a Leica DM 1000 microscope.

#### Caco-2 Cell Culture Adhesion Assay

The Caco-2 enterocyte cell line was obtained from the cell bank at the CIB. They were seeded and maintained as previously described (Garai-Ibabe et al., 2010b). For adhesion assays, exponential-phase cultures of the *P. ethanolidurans* strains after sedimentation were resuspended in DMEM medium (Invitrogen), to give a concentration of 1.25 × 10^6^ cfu/mL, and added to Caco-2 cells (ratio 10:1, bacteria:Caco-2 cells) in a final volume of 0.1 mL per well. After incubation for 1 h at 37°C under a 5% CO_2_ atmosphere, unattached bacteria were removed by three washes with 0.2 mL of PBS pH 7.2. Then, the Caco-2 cells were detached from the plastic surface by addition of 0.1 mL of 0.05% trypsin–EDTA per well and incubation for 5 min at 37°C. The detachment was stopped by adding 0.1 mL of PBS pH 7.2. To determine the number of cell-associated bacteria, appropriate dilutions were plated onto MRS-agar plates. The experiments were performed in triplicate. Adhesion data were analyzed by two-way analysis of variance (ANOVA) to determine the significant differences between the variables at *p* ≤ 0.05. The analysis was performed using the SAS 9.4 software (SAS Institute Inc., Cary, NC, United States).

## Results and Discussion

### *P. ethanolidurans* CUPV141 Produces a HoPS

The mucosal (ropy) phenotype of some bacteria is related to the production of EPS (Torino et al., 2015), and among others, we have previously isolated the 2-substituted (1,3)-β-D-glucan-producing *P. parvulus* 2.6 strain from cider due to its ropy phenotype (Fernández et al., 1995). In the search for novel β-D-glucan-producing bacteria, the CUPV141 strain was isolated from a ropy cider and selected by its mucosal phenotype upon growth in a medium containing glucose. Determination of the nucleotide sequence of the 16S RNA coding gene (accession number in GenBank: MH298647) identified this strain as *P. ethanolidurans* and, as far as we know, this is the first instance of isolation of this species from a ropy cider.

**FIGURE 1:**
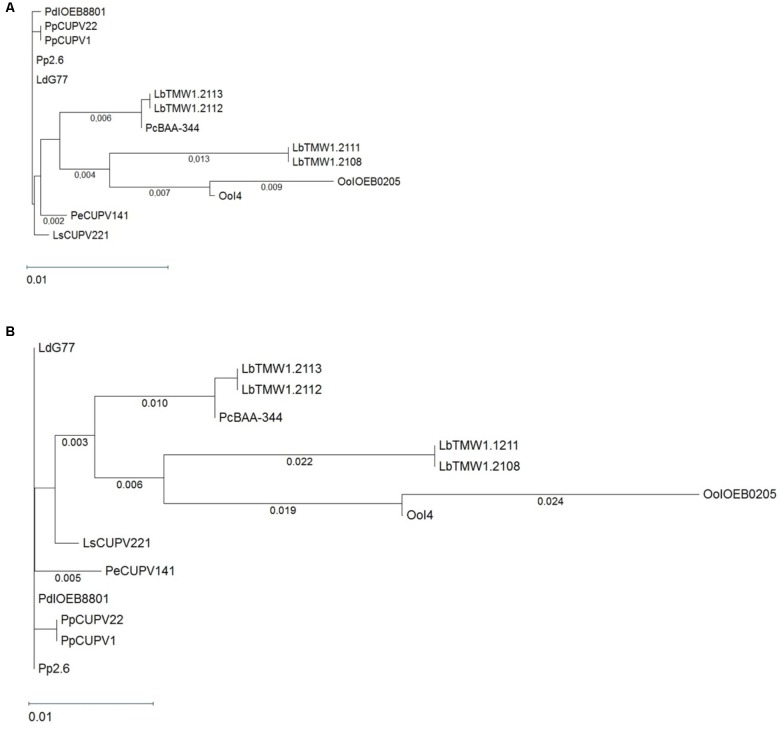
Phylogenetic trees pertaining to **(A)** the *gtf* genes and **(B)** the GTF protein of different lactic acid bacteria. The trees were obtained with the Tamura and Nei (1993) and Kimura metrics, for DNA and proteins, respectively, from a MSA generated with the Clustal Omega algorithm. Complete names and details of the strains are depicted in **Table 1**.

A specific ELISA method developed in our group (Werning et al., 2014) was used to test and quantify the production of the 2-substituted (1,3)-β-D-glucan by *P. ethanolidurans* CUPV141 cultures (OD_600__nm_ = 1.0), which amounted to 59.8 ± 4.8 mg/L. Afterwards, we proceeded to characterize the genetic determinant responsible for the β-D-glucan synthesis, which is synthetized by the GTF enzyme. Previously designed oligonucleotides (Werning et al., 2006) were used to amplify the *gtf* gene of CUPV141, and to determine its nucleotide sequence (accession number in GenBank: MH028492), which was compared to those of the *gtf* genes from GenBank at the NCBI site. The BLASTn analysis revealed a 99% identity of the CUPV141 *gtf* with its homologs from *Lactobacillus suebicus* CUPV221, *Lactobacillus diolivorans* G77 (CUPV218) and *P. parvulus* CUPV22, CUPV1 and 2.6 strains isolated from Spanish cider and from *P. damnosus* IOEB8801 isolated from French wine, showing that these genes have evolved from a common ancestor. Thirteen *gtf* nucleotide sequences encoded by several LAB, belonging to the *Pediococcus, Lactobacillus*, and *Oenococcus* genera, were compared to that of CUPV141 strain to obtain the MSA depicted in **Supplementary Figure S1** and the phylogenetic unrooted tree shown in **Figure 1A**. According to the phylogenetic tree, the *gtf* gene is highly conserved among the species and genera studied, although the MSA showed some nucleotide changes. Also, a high identity was observed between the *gtf* genes of *P. ethanolidurans* CUPV141 and *P. parvulus* 2.6, the reference bacterium for this gene. Only four changes, located at positions 86, 217, 1291, and 1524, differentiate these strains. The last was a silent mutation and the other three resulted in changes of the GTF of *P. parvulus* 2.6 (Trp29Leu, Leu73Phe, and H43Y) Thus, two divisions can be made according to the origin from which the species were isolated. The bacteria isolated from cider and *P. parvulus* IOEB8801 (from wine) are grouped together. All of them contain a plasmid harboring the *gtf* gene, except *L. suebicus* CUPV221 for which the location of the gene has not been established. The second division mainly grouped isolates from beer, besides two *O. oeni* strains isolated from champagne and cider, both described as having the *gtf* gene at a chromosomal localization. Taking into consideration this classification, there are also some mutations to emphasize. For instance, those having changes at positions 272, 1524, or 1548, where the majority of *gtf* genes of bacteria isolated from cider harbor two cytosines and one adenine, while the genes of beer isolates carry two thymines and one guanine, respectively. Moreover, the mutation in position 272 (a change Ala91Val) only occurred in the GTF of the isolates from cider. The other two were silent mutations. Although the physiological role of the 2-substituted (1,3)-β-D-glucan production is unknown, the high conservation of the *gft* gene could be due to a bacterial adaptation to the alcoholic environment of the different beverages. The plasmidic location of the gene in the *Pediococcus* and *Lactobacillus* strains suggests a horizontal transfer of the gene, which might have conferred an evolutionary advantage.

**Table 1:** Bacterial strains used for the *gtf* MSA and their codes.

Code	Strain	*gtf* gene location	Source of isolation	Reference
***Pediococcus***		
PeCUPV141	*P. ethanolidurans* CUPV141	Plasmid pPE3	Cider	This work
Pp2.6	*P. parvulus* 2.6	Plasmid pPP2	Cider	Werning et al., 2006
PpCUPV1	*P. parvulus* CUPV1	Plasmid	Cider	Garai-Ibabe et al., 2010a
PpCUPV22	*P. parvulus* CUPV22	Plasmid	Cider	
PdIOEB8801	*P. damnosus* IOEB8801	Plasmid pF8801	Red wine	Gindreau et al., 2001
PcBAA-344	*P. claussenii* ATCC BAA-344	Plasmid	Beer	Pittet et al., 2012
***Oenococcus***		
OoI4	*O. oeni* I4	Chromosome	Cider	Werning et al., 2006
OoIOEB0205	*O. oeni* IOEB205	Chromosome	Champagne	Dols-Lafargue et al., 2008
***Lactobacillus***		
LsCUPV221	*L. suebicus* CUPV221	Unknown	Cider	Garai-Ibabe et al., 2010b
LdG77	*L. diolivorans* G77 = CUPV218	Plasmid pLD1	Cider	Werning et al., 2006
LbTMV1.2108	*L. brevis* TMV1.2108	Plasmid pl12108-6	Beer	Fraunhofer et al., 2018
LbTMV1.2111	*L. brevis* TMV1.2111	Plasmid pl12111-5	Beer	Fraunhofer et al., 2018
LbTMV1.2112	*L. brevis* TMV1.2112	Plasmid pl12112-4	Beer	Fraunhofer et al., 2018
LbTMV1.2113	*L. brevis* TMV1.2113	Plasmid pl12113-4	Brewery surface	Fraunhofer et al., 2018

The translated *P. ethanolidurans* CUPV141 *gtf* gene was used as a template for a BLASTp search. Most of the high identity hits coincided with those detected for the GTF enzymes, but the search also revealed identities, not detected at the nucleotide level, such as the GTF from *Propionibacterium freudenreichii* (33%) or the Tts glycosyltransferase of *S. pneumoniae* (36%). These results indicate a convergent functional evolution to yield glycosyltransferases encoded from unrelated genes. Similar results were reported for the GTF of *S. pneumoniae* Tts and *Propionibacterium freudenreichii* (Deutsch et al., 2008), and for those of *P. parvulus* 2.6, *P. damnosus* IOEB8801, and *O. oeni* I4 (Werning et al., 2006; Dols-Lafargue et al., 2008).

Moreover, the amino acid sequences of CUPV14 and 13 glycosyltransferases, encoded by related genes (**Figure 1A**) were aligned (**Supplementary Figure S2**). A phylogenetic tree was also generated (**Figure 1B**) disclosing a high degree of conservation, with small evolutionary distances among the GTF of different LAB species. Again, the species clustered according to the source from which they were isolated, with a clear grouping of the isolates from cider and beer, and high similarities in the active center of all the enzymes compared.

**FIGURE 2:**
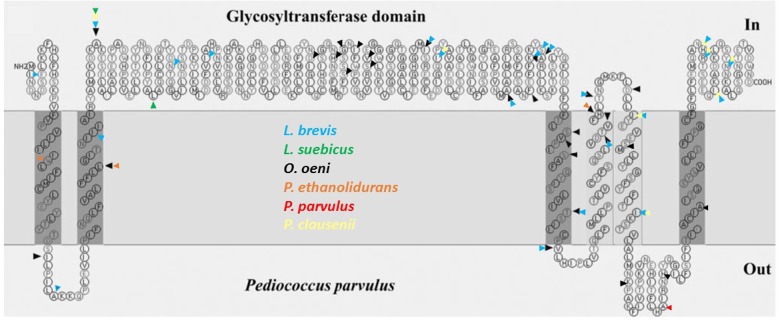
Mutations of different lactic acid bacteria in the protein sequence of the GTF, pertaining to the secondary structure predicted for *P. parvulus* 2.6 and *L. diolivorans* G77 (CUPV218) using the SOSUI program (http://harrier.nagahama-i-bio.ac.jp/sosui/). Each colored arrowhead represents a bacterial species: black for *O. oeni*, blue for *L. brevis*, green for *L. suebicus*, yellow for *Pediococcus clausenii*, orange for *P. ethanolidurans* and red for *P. parvulus*.

In addition, the differences in amino acids of the fourteen glycosyltransferases were assembled (**Figure 2**) in a previous topological prediction of the GTF of *P. parvulus* 2.6 and *L. diolivorans* G77 (CUPV218) (Werning et al., 2006). According to this model, the translated polypeptide seems to have four transmembrane regions at the C-terminal domain and two more at its N-terminus flanking the catalytic domain. This suggests that the enzyme synthesizes the EPS in the cytosol and that the active protein could be an oligomer of the polypeptide, which could form a membrane pore through which the polymer could be secreted to the environment. The alignment (**Supplementary Figure S2**) and superimposition (**Figure 2**) of the amino acid sequences revealed that the main differences are located at the transmembrane regions, which could be explained as an adaptation for optimal insertion into the membrane of each particular bacterium. Some variations were also observed in the sequence of the active center of the proteins, especially in *O. oeni* IOEB0205, but none of them affected the aspartic acid residues (Asp143, Asp198 and Asp200 or Asp295) that seem to constitute the essential catalytic tetrad (Werning et al., 2012). The GTF of *P. parvulus* CUPV1 and CUPV22 only differ with the enzyme of *P. parvulus* 2.6 in one amino acid (T489A), located at the loop between the fifth and sixth predicted transmembrane regions (Garai-Ibabe et al., 2010b). Finally, the four nucleotide mutations present in CUPV141 *gtf* gene resulted in three changes in the encoded polypeptide, L29W, F73L and Y431H, highlighted in orange in **Figure 2** and located at the first, second, and fourth predicted transmembrane regions, respectively, and the fourth change in the nucleotide sequence in position 1524 resulted in a silent mutation.

### Isolation and Partial Characterization of the EPS From *P. ethanolidurans* CUPV141

Culture media for routine growth often contain components that interfere with the quantification of the EPS released to the medium by bacteria, i.e., MRS medium (De Man et al., 1960). For this reason, SMD medium was used for the isolation of the EPS produced by CUPV141. As reported before (Velasco et al., 2006), the production of EPS can be affected by growth conditions, as well as by the growth media. Therefore the bacterial strain was cultivated for 48 h at two different pHs in SMD medium, giving a slightly higher production of EPS when the medium was adjusted to pH 5.5 (58.9 ± 2.2 mg/L) rather than pH 4.8 (53.5 ± 1.2 mg/L). Thus, for the subsequent isolation of EPS from the supernatants, both *Pediococcus* strains were grown in SMD medium at pH 5.5 for 48 h at 28°C in an atmosphere containing 5% CO_2_.

The yield of EPS recovered from the supernatant of CUPV141 was 69 mg per liter of culture. Among the cider isolates, *P. parvulus* CUPV1 and *L. suebicus* CUPV221 produced similar amounts of EPS, while *P. parvulus* 2.6 or *P. parvulus* CUPV22 have been reported to produce 193 and 243 mg per liter, respectively (Garai-Ibabe et al., 2010a).

In order to elucidate the partial chemical structure of the EPS, several analyses were carried out. The polymer released to the medium by CUPV141 contained glucose as the major monosaccharide, and small amounts of galactose and galactosamine were also detected (less than 5%). In addition, a peak was identified as glycerol-3-phosphate using the NIST library included in the chromatographic software, which is an uncommon component of EPS from *Pediococcus* strains.

**FIGURE 3:**
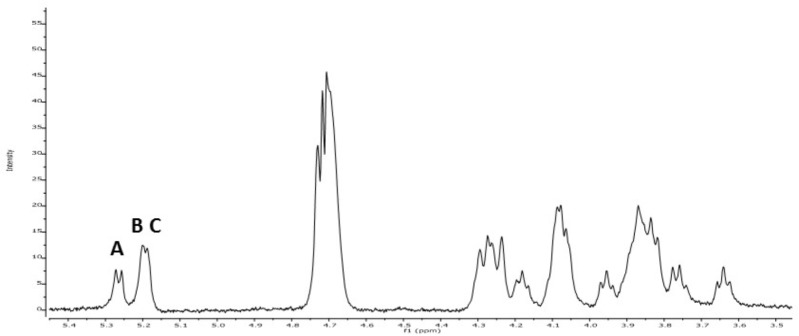
^1^H RMN spectra of CUPV141 EPS recovered from the supernatant. A, B, and C signals correspond to the anomeric protons of terminal β-D-glucopyranose units, 3-linked- β-D-glucopyranose residues and 2,3-linked- β-D-glucopyranose units, respectively, as described in Dueñas-Chasco et al. (1997) for the 2-substituted (1,3)-β-D-glucan produced by *P. parvulus* 2.6.

Methylation analysis gave evidence of three main units of partially methylated alditol acetates corresponding to terminal, 3-*O*-substituted and 2,3-di-*O*-substituted glucopyranose in relative proportions 1:1:1. In addition, the ^1^H NMR spectrum of the EPS (**Figure 3**) showed the signals reported for the 2-substituted (1,3)-β-D-glucan of *P. parvulus* 2.6 (Dueñas-Chasco et al., 1997), which confirmed that *P. ethanolidurans* CUPV141 releases this β-D-glucan as the major extracellular polysaccharide. However, other minor components were identified in this EPS: (1,2)-galactopyranose, (1,6) and (1,3,6)-glucopyranose, and (1,4)-glucosamine, suggesting that *P. ethanolidurans* CUPV141 produced also a HePS. To the best of our knowledge, the production of both a HoPS and a HePS by *Pediococcus* strains has not been previously reported.

### The *P. ethanolidurans* CUPV141NR Mutant Strain

**FIGURE 4:**
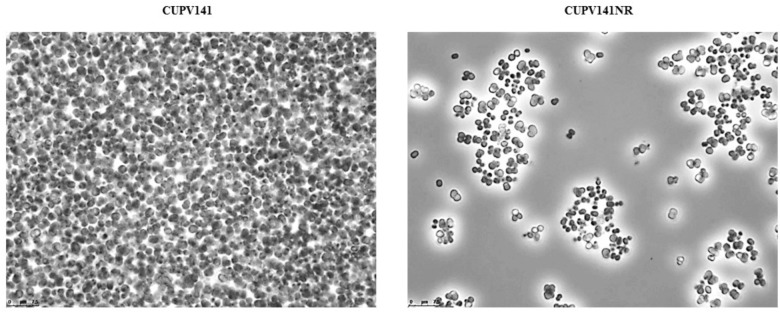
Immunoagglutination in the presence of anti-serotype 37 antibody of (1,3)(1,2)-β-D-glucan producing *P. ethanolidurans* strains CUPV141 and CUPV141NR.

Chemical mutagenesis of *P. parvulus* 2.6 resulted in the generation of the 2.6NR isogenic strain that did not produce β-D-glucan (Fernández et al., 1995). Therefore, with the aim of abolishing the production of the HoPS for further studies on the biological activity of the EPS, and for a better characterization of the HePS synthesized, *P. ethanolidurans* CUPV141 was subjected to chemical mutagenesis and the isogenic non-ropy CUPV141NR strain was generated. The Tts glycosyltransferase of *S. pneumoniae* serotype 37, which is homologous to the GTF of *P. parvulus* 2.6 and *P. ethanolidurans* CUPV141, synthesizes a capsular HoPS (Llull et al., 1999) very similar to the β-D-glucan produced by the pediococcal enzyme. Thus, anti-serotype 37 antibodies are able to agglutinate 2-substituted (1,3)-β-D-glucan-producing bacteria (Llull et al., 1999; Werning et al., 2006). Therefore, an evaluation of the HoPS production of the mutant and the parental strain by an agglutination immunoassay with anti-serotype 37 antibodies was performed. The results revealed a clear difference between the aggregation capabilities of the two strains, showing, after a 24 h-incubation period, that *P. ethanolidurans* CUPV141 formed huge aggregates in the presence of the antibodies, while CUPV141NR strain produced small aggregates (**Figure 4**). These results suggested that the mutant strain still produced the β-D-glucan, although at very low levels. Specific quantification of the 2-substituted (1,3)-β-D-glucan with the ELISA immunoassay using anti-serotype 37 antibodies confirmed that, at an OD_600__nm_ = 1.0, the non-ropy CUPV141NR strain released 0.096 ± 0.002 mg/L of β-glucan to the supernatant, whereas the wild-type CUPV141 strain produced 42.40 ± 0.05 mg/L.

The recovery of EPS from the supernatant of CUPV141NR yielded 29 mg per liter of culture, a quantity much lower than that produced by the ropy strain. Acid hydrolysis of the EPS released glucose, galactose and glucosamine in a molar ratio 2.4:1:0.9, and the peak of glycerol-3-phosphate was observed again in the chromatogram. The main linkage types in the EPS, deduced from a methylation assay, revealed a polymer structurally different from the major β-glucan produced by the ropy strain, but with the same components detected in minor amounts in that preparation, namely: *O*-2 substituted galactopyranose, terminal glucopyranose, *O*-6, *O*-2,6, and *O*-3,6 substituted glucose, and *O*-4 substituted glucosamine. The proportion of terminal residues was far lower than that expected from the amount of branching points, which suggests that the glycerol 3-phosphate units detected in the hydrolysates may occupy terminal positions in the side chains of this branched polymer. Further analyses are needed to determine the structure of this polymer, but the current data confirm that *P. ethanolidurans* CUPV141 synthetizes and secretes at least two polysaccharides: the 2-substituted (1,3)-β-D-glucan and a HePS with glycerol-3-phosphate. This is the first instance of a *Pediococcus* strain producing both a HoPS and a HePS, and the first report of a phosphorylated EPS in this genus, although other HePS with phosphorylated glycerol have been reported in *Lactobacillus delbrueckii* ssp. *bulgaricus* OLL 1073R-1 (Kitazawa et al., 1998), *Lactobacillus plantarum* EP56 (Tallon et al., 2003), and *Lactobacillus johnsonii* FI9785 (Dertli et al., 2013).

### Detection of the Plasmidic Location of *P. ethanolidurans gtf* Gene

**FIGURE 5:**
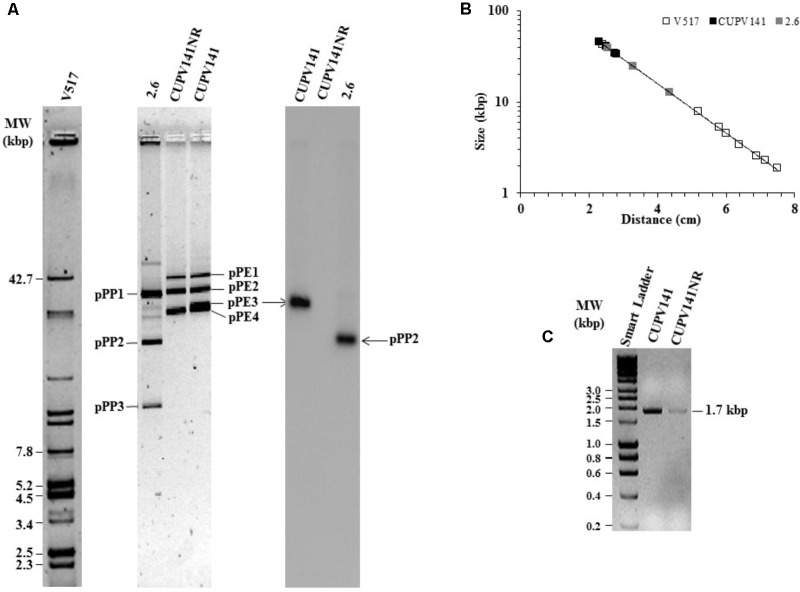
Detection of plasmids of *P. ethanolidurans* CUPV141 and CUPV141NR strains and of *P. parvulus* 2.6. **(A)** Detection of the *gtf* gene by Southern blot hybridization. Left, analysis in a 0.7% agarose gel of plasmids preparations of LAB strains and of *E. coli* V517. Right, hybridized membrane of samples transferred from the agarose gel. **(B)** Depicts the calibration curve for plasmid size determination. Symbols: plasmids from *E. coli* V517 (◊), *P. ethanolidurans* (♦) and *P. parvulus* (♦) strains. **(C)** Analysis in 0.7% agarose gel of *gtf* PCR amplicons obtained with genomic DNA from CUPV141 and CUPV141NR strains. Smart Ladder, molecular weight standard.

The *gtf* gene of *P. parvulus* 2.6 is located in the pPP2 plasmid, which is not present in the 2.6NR strain (Fernández et al., 1995; Werning et al., 2006; Pérez-Ramos et al., 2017b). Thus, by homology, the *gtf* gene in *P. ethanolidurans* CUPV141 could be located in a plasmid, and the reason for the non-ropy phenotype of the isogenic CUPV141NR strain could be the loss of this plasmid. To confirm this hypothesis, total plasmid DNA preparations from *P. ethanolidurans* CUPV141, CUPV141NR and *P. parvulus* 2.6 were purified by fractionation in a CsCl gradient to eliminate open circles and linear forms of the plasmids. Then, the purified plasmid DNA preparations were fractionated in an agarose gel (**Figure 5A**). As expected, three bands were detected in the preparation of *P. parvulus* 2.6 corresponding to the previously identified pPP1, pPP2, and pPP3 plasmids with molecular weights of 39.1, 24.5, and 12.7 kbp, respectively (Pérez-Ramos et al., 2017a and molecular weight inferred in **Figure 5B**). A different plasmidic pattern was detected in the *P. ethanolidurans* CUPV141 DNA preparation, including four bands, which should correspond to plasmids named pPE1, pPE2, pPE3, and pPE4 with an inferred molecular weight of 45.6, 40.2, 34.4, and 33.4 kbp, respectively (**Figure 5B**). Only three bands were observed in the preparations of the *P. ethanolidurans* CUPV141NR strain, which lacked the pPE3 plasmid. Southern blot hybridization gave evidence of the presence of the *gtf* gene in *P. parvulus* 2.6 and in the *P. ethanolidurans* wild-type strain and not in the mutant (**Figure 5A**). Moreover, the hybridization bands revealed that pPE3 harbors the *gtf* gene in CUPV141, whose molecular weight differs from that of the pPP2 *gtf*-carrier plasmid in *P. parvulus* 2.6. The non-detection of *gtf* in the CUPV141NR strain correlated with its non-ropy phenotype, however, as stated above, the immunodetection and specific quantification of the (1,3)-β-D-glucan indicated that this bacterium produces low levels of the HoPS. This could be due to the presence of pPE3 in CUPV141NR with a low copy number undetectable by Southern blot hybridization. Thus, plasmidic DNA from both *Pediococcus* strains was used for the detection of the *gtf* gene by the more sensitive PCR amplification method. The reaction products were analyzed in agarose gel (**Figure 5C**), and the analysis revealed that the expected amplicon of 1.7 kbp had been generated with both plasmidic DNA preparations. However, the intensity of the band was very weak in the CUPV141NR DNA preparation even though a fivefold higher reaction volume, compared to that of CUPV141 DNA, was loaded in the gel (**Figure 5C**). Consequently, the overall results support a drastic decrease of the copy number of pPE3 as a consequence of the novobiocin treatment of CUPV141 to generate the CUPV141NR strain.

### Genetic Determinant of the Initiation of HePS Synthesis in *Pediococcus* Strains

**FIGURE 6:**
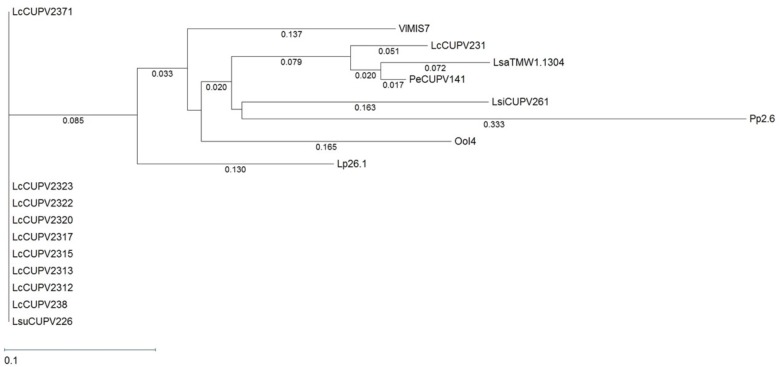
Phylogenetic tree pertaining to the *p-gtf* gene nucleotide sequences of different lactic acid bacteria. The tree was obtained with the Tamura and Nei (1993) metric from a MSA generated with the Clustal Omega algorithm. Complete names and details of the strains are depicted in **Table 2**.

HePS are synthesized by a more complex molecular mechanism than HoPS, which requires the action of several proteins. It is known that the first enzyme in the process is the p-GTF, which transfers the first phospho-sugar residue from an activated nucleotide sugar to the undecaprenyl phosphate-lipid carrier embedded in the membrane (Lebeer et al., 2009). The pGTF from *O. oeni* has been recently characterized (Dimopoulou et al., 2017), but no studies in *P. ethanolidurans* have been performed until now. Thus, the detection of the p-GTF coding gene was approached by PCR amplification of the highly conserved C-terminal sugar transferase domain of the enzyme, using degenerate primers previously described (Provencher et al., 2003). The nucleotide sequence of the obtained fragments was the same for the ropy and non-ropy strains. Furthermore, BLASTn analysis revealed 96, 85, and 74% identities with regions of genes encoding putative proteins annotated as a sugar transferase of *Lactobacillus sanfranciscensis* TMV1.1304, a glycosylphosphotransferase of *Vagococcus lutrae* MIS7, and a p-GTF of *Lactobacillus plantarum* 26.1, respectively. These nucleotide sequences were aligned using the Clustal Omega algorithm (**Supplementary Figure S3**) and a phylogenetic tree was also obtained (**Figure 6**). In addition, the MSA included a DNA sequence of a gene encoding a putative undecaprenyl-phosphate galactosephosphotransferase from the genome of *P. parvulus* 2.6 (Pérez-Ramos et al., 2016a), and the partial DNA sequences of the *p-gtf* genes from different bacteria isolated from cider in a previous work (Puertas et al., 2018). The MSA showed a very poor conservation of the gene between different species. For instance, *P. parvulus* 2.6 showed three triplets more than the rest of the bacteria at position 125. However, the homology was very high among different strains of *Lactobacillus collinoides*, as is also observed in the phylogenetic tree, except for the CUPV231 strain, which has an identity of 63.4% (104 out of 164 nucleotides) with the other *L. collinoides p-gtf* analyzed. The evolutionary distances between different species, which are depicted in the tree, would confirm this fact, being inexistent between *L. collinoides* strains. For the CUPV141 *p-gtf* DNA fragment, the amino acidic sequence of the encoded polypeptide was inferred with the EditSeq program and it was subjected to BLASTp analysis, where various hits were found. The amino acid sequences, like their corresponding coding ones, showed a high degree of identity among different strains of the same species, and less conservation between different species (**Supplementary Figure S4**). The C-terminal region of the LAB p-GTFs includes two blocks, B and C related, respectively, to either the interaction with the lipid carrier or conferring the specificity for sugar recognition (Wang et al., 1996). All of the amino acid sequences aligned have a Glu at position 5 of block C except for that of *P. parvulus* 2.6, which carries a Phe (**Supplementary Figure S4**), previously proposed to be a catalytic residue (Van Kranenburg et al., 1999). In addition, two Tyr present in the C block had been proposed to be implicated in the phosphorylation of the enzyme in *S. thermophilus* (Minic et al., 2007). The one located at position 9 of the block is present in all of the sequences except for that of the 2.6 strain, which carries a phenylalanine (F), substitution that has no effect in the *S. thermophilus* p-GTF (Minic et al., 2007). In addition, an *in silico* analysis to find the genomic location of the *P. parvulus* 2.6 *p-gtf* gene revealed that it is included in a cluster of 11 genes involved in HePS synthesis and secretion (**Supplementary Figure S5**). Therefore, production of HePS by *Pediococcus* does not seem to be limited to the *P. ethanolidurans* species. However, we have never detected synthesis of HePS by the 2.6 strain and this could be due to lack of functionality of its p-GTF.

**Table 2:** Bacterial strains used for the *p*-*gtf* MSA and their codes.

Code	Strain	Source of isolation	Reference
PeCUPV141	*P. ethanolidurans* CUPV141	Cider	This work
Pp2.6	*P. parvulus 2.6*	Cider	Pérez-Ramos et al., 2016a
Lp26.1	*L. plantarum* 26.1	Dairy and cereals	Van der Meulen et al., 2007
LsaTMV1.1304	*L. sanfranciscensis* TMV1.1304	Sourdough	Vogel et al., 2011
LsiCUPV261	*L. sicerae* CUPV261	Cider	Puertas et al., 2018
LsuCUPV226	*L. suebicus* CUPV226	Cider	Puertas et al., 2018
LcCUPV238	*L. collinoides* CUPV238	Cider	Puertas et al., 2018
LcCUPV2312	*L. collinoides* CUPV2312	Cider	Puertas et al., 2018
LcCUPV2313	*L. collinoides* CUPV2313	Cider	Puertas et al., 2018
LcCUPV2315	*L. collinoides* CUPV2315	Cider	Puertas et al., 2018
LcCUPV2317	*L. collinoides* CUPV2317	Cider	Puertas et al., 2018
LcCUPV2320	*L. collinoides* CUPV2320	Cider	Puertas et al., 2018
LcCUPV2322	*L. collinoides* CUPV2322	Cider	Puertas et al., 2018
LcCUPV2323	*L. collinoides* CUPV2323	Cider	Puertas et al., 2018
LcCUPV2371	*L. collinoides* CUPV2371	Cider	Puertas et al., 2018
LcCUPV231	*L. collinoides* CUPV231	Cider	Puertas et al., 2018
OoI4	*O. oeni* I4	Cider	Puertas et al., 2018
VlMIS7	*Vagococcus lutrae* MIS7	Fermented food	Unpublished, GenBank: AGM39429.1

### Analysis of the Adhesion Ability of the *P. ethanolidurans* CUPV141 and CUPV141NR Strains

To carry out this test, bacteria have to be sedimented and thus the supernatant containing part of the EPS is removed. Therefore, the concentration of the 2-substituted (1,3)-β-D-glucan bound to the bacteria after sedimentation and resuspension was determined by the specific ELISA immunoassay method prior to the analysis. As expected, the results revealed differences between the two strains, since CUPV141 and CUPV141NR carry 80.2 ± 6.0 ng/mL and <0.30 ± 0.06 ng/mL HoPS, respectively. Consequently, a comparative analysis of the two strains analyzed should provide information on the contribution of the HoPS to adhesion.

**FIGURE 7:**
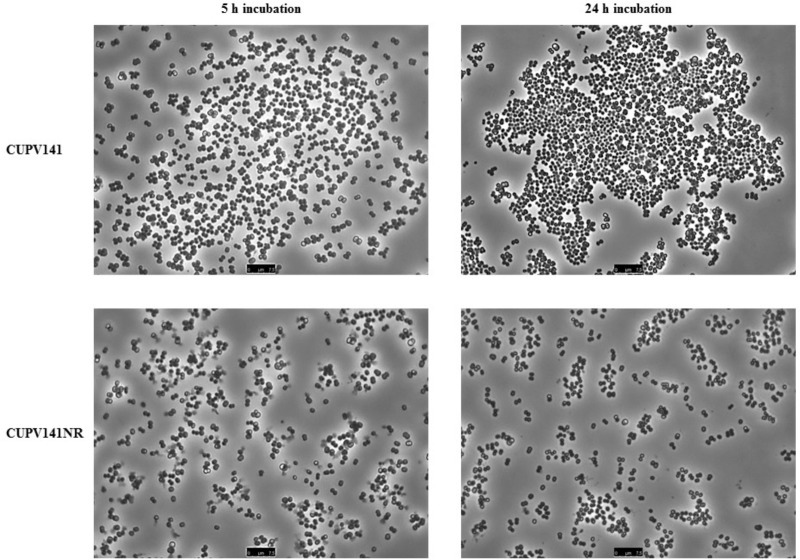
Aggregation of *P. ethanolidurans* CUPV141 and CUV141NR strains, incubated for 5 h and 24 h.

First, the ability of the bacteria for self-aggregation was investigated (**Figure 7**). After 5 h of incubation, the culture of *P. ethanolidurans* CUPV141 showed some aggregates, whereas fewer complexes appeared in the culture of CUPV141NR. However, the differences at this incubation time were not very appreciable. By contrast, after 24 h of incubation the aggregates formed by the CUPV141 strain were bigger than those in the 5-h culture. An increase of aggregation of the CUPV141NR strain was not observed for the 5 h to 24 h incubation period. Thus, these results indicated that bacterial cell-to-cell interactions are mediated or potentiated by the 2-substituted (1,3)-β-D-glucan.

**FIGURE 8:**
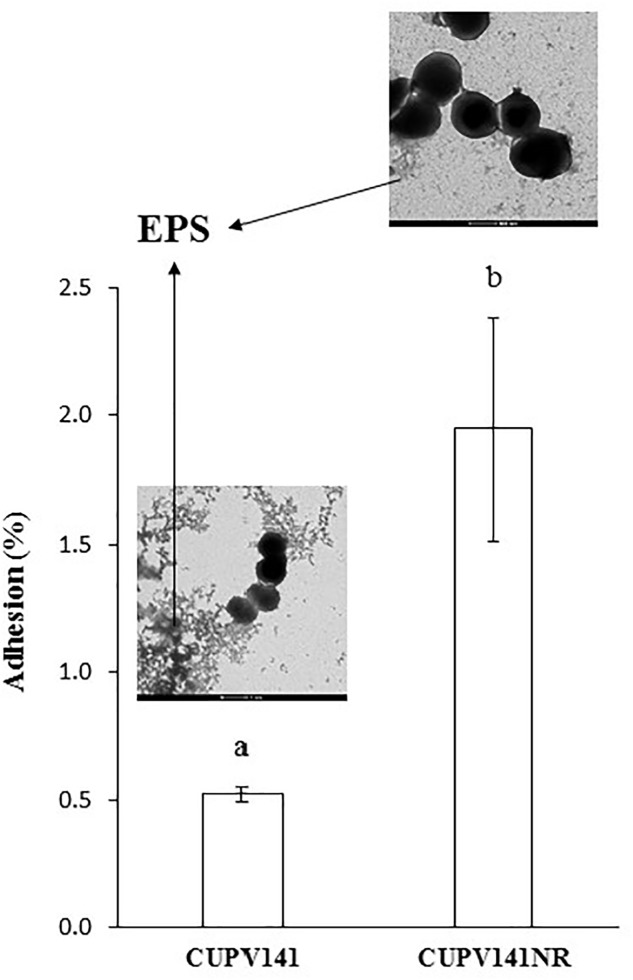
Adhesion of *P. ethanolidurans* CUPV141 and CUPV141NR strains to Caco-2 cells. Values are expressed as the percentage of cfu added to the assay. The results are the mean of three independent experiments. The insets show electron micrographs of the bacteria. The arrows mark the β-glucan (EPS). Statistical significances are represented by different letters that mean a *p* ≤ 0.05.

Secondly, the capacity of *P. ethanolidurans* CUPV141 and CUPV141NR to interact with human epithelial cells was also assessed using the enterocyte-like Caco-2 cell line (**Figure 8**). Visualization of the two strains by TEM (inset in **Figure 8**) showed differences in EPS production, which in CUPV141 formed a kind of net in the medium, while in CUPV141NR appeared as small aggregates. The ability of both strains to bind the enterocytes was significantly lower than that reported by Fernández De Palencia et al. (2008) for the commercial probiotic *Lactobacillus acidophilus* La-5 (7%). However, comparing the two strains described in this work, the non-ropy bacterium showed higher adhesion (1.95 ± 0.44%) to the eukaryotic cells than the ropy strain (0.52 ± 0.03%), which can be attributed to the presence of the phosphorylated HePS. Phosphate groups increase the net charge of the EPS and can be very important to mediate the interactions between bacteria and their hosts, as reported for several *Lactobacillus* strains (Kitazawa et al., 1998; Tallon et al., 2003; Dertli et al., 2013). Similarly, chemical phosphorylation of a dextran produced by *Leuconostoc mesenteroides* enhanced its immunostimulatory capacity (Sato et al., 2004). For neutral EPS, some authors have reported that their presence in the surface of bacteria has a negative effect in their adhesive properties (López et al., 2012; Castro-Bravo et al., 2017; Nácher-Vázquez et al., 2017), while others described the production of these polymers as useful for probiotics to interact with eukaryotic cells (Fernández de Palencia et al., 2009; Garai-Ibabe et al., 2010b; Živković et al., 2016).

The positive effect of the HoPS on binding to intestinal cells was demonstrated for the β-glucan-producing *P. parvulus* 2.6 and CUPV22 strains, which showed adhesion capacities to Caco-2 cells of 6.1 and 10.5%, respectively, that were reduced when the EPS was removed by washing prior to the binding assay (Fernández de Palencia et al., 2009; Garai-Ibabe et al., 2010b). Thus, the different behavior of the CUPV141 ropy strain could be due to its lower production of 2-substituted (1,3)-β-D-glucan. The overall results obtained for *P. ethanolidurans* illustrate the different roles of the two polysaccharides produced by this species: there is an involvement of the 2-substituted (1,3)-β-D-glucan in cell-to-cell adhesion, while the HePS would lead these bacteria to interact with eukaryotic cells, for colonization of new environments. However, further research would be necessary for the elucidation of the mechanism through which these kinds of adhesions take place.

## Conclusion

Bacterial EPS (especially from LAB) are currently exploited in the food and beverage industries as the fermented products have improved texture and flavor. There is evidence that consumption of such products can have health benefits. This in turn has led to research to specifically identify EPS that could be potentially developed as medicines for human and animal use (Castro-Bravo et al., 2017; Dimopoulou et al., 2018; Pérez-Ramos et al., 2017a,b, 2018). Therefore, in the search for novel bacterial producers of the extracellular 2-substituted-(1,3)-β-D-glucan, we isolated a *P. ethanolidurans* (CUPV141) strain for the first time from a ropy cider of the Basque Country. In this work, we demonstrate that this isolate secretes not only that β-glucan, but also a HePS composed of glucose, galactose, glucosamine, and glycerol-3-phosphate, being the first *Pediococcus* strain described to produce this kind of polymer. However, our *in silico* analysis of priming-glycosyltransferase coding genes involved in HePS synthesis suggests that this is a general characteristic shared by different pediococci. Southern blot hybridization allowed localizing the GTF-coding gene responsible for the synthesis of the β-D-glucan in a 34.4 kbp-pPE3 plasmid of this strain. The role of the HoPS in bacterial self-aggregation, as well as the most relevant role of the HePS in bacteria-eukaryotic cells interactions were inferred from interactomic experiments using the ropy and the non-ropy strains. Nevertheless, the molecular mechanisms by which *P. ethanolidurans* performs biotic interactions, the detailed structure of the HePS produced by this strain, and its existence in other pediococci remain unknown and deserve further work.

## Data Availability

All datasets generated for this study are included in the manuscript and the Supplementary Files.

## Author Contributions

ML-A was responsible for the manuscript’s preparation and performed the majority of the assays described. AP-R was responsible for the extraction of the plasmids and the performance of the southern-blot hybridization assay. AP was responsible for the strain identification and determination of the DNA sequences of the priming-GTF coding gene from different LAB isolated from cider. AP was responsible for the planning of the experimental work and for the performance of the methylation and monosaccharide composition analysis in the characterization of the EPS. In addition, she participated in the manuscript corrections. PL and MD contributed to the discussions of the results, had a critical reading of the manuscript and corrected it.

## Conflict of Interest Statement

The authors declare that the research was conducted in the absence of any commercial or financial relationships that could be construed as a potential conflict of interest. The handling editor declared a past co-authorship with several of the authors AP-R and PL.

## Footnotes

1 https://www.ezbiocloud.net

2 http://www.ncbi.nlm.nih.gov/

3 https://blast.ncbi.nlm.nih.gov/
