# Supplementary material for: Characterization of Pediococcus ethanolidurans CUPV141: A β-D-glucan- and Heteropolysaccharide-Producing Bacterium
Source: Front Microbiol. 2018 Sep 4;9:2041. doi: 10.3389/fmicb.2018.02041 (PMC6131198; doi:10.3389/fmicb.2018.02041)
Supplement: Supplementary file 1 [file Table_1.docx]

Supplementary Material

**Characterization of *Pediococcus ethanolidurans* CUPV141: a β-D-glucan- and heteropolysaccharide-producing bacterium**

María Goretti Llamas-Arriba^1,2^, Adrián Pérez-Ramos^2^, Ana Isabel Puertas^1^, Paloma López^2^, María Teresa Dueñas^1^ and Alicia Prieto^2*^

*** Correspondence:** Corresponding Author: Alicia Prieto Orzanco; aliprieto@cib.csic.es


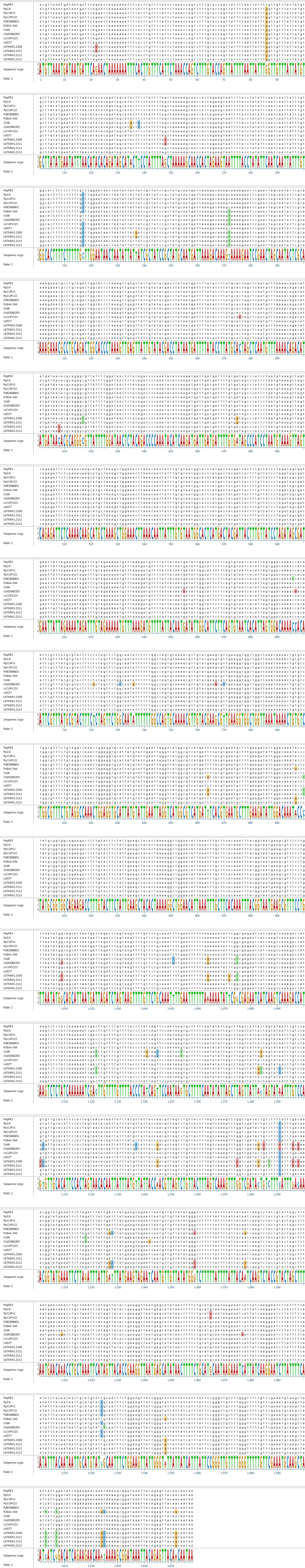


**
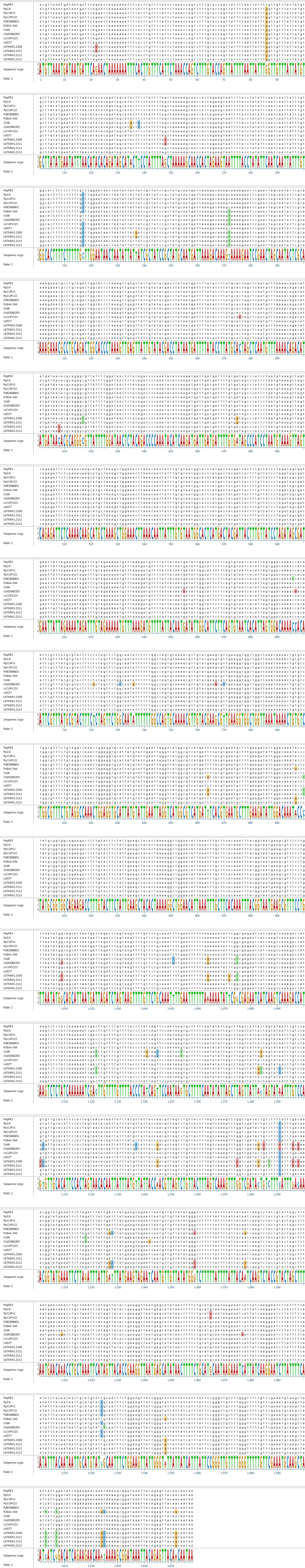
**

**
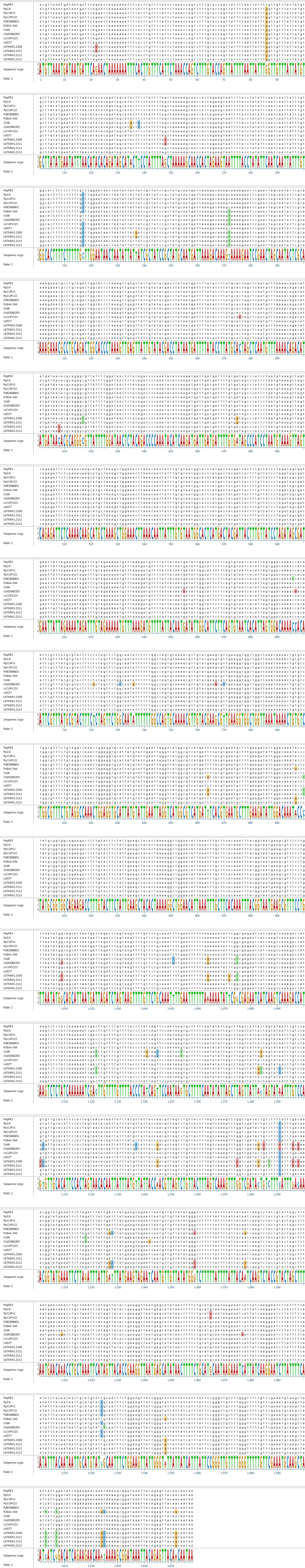
**

**
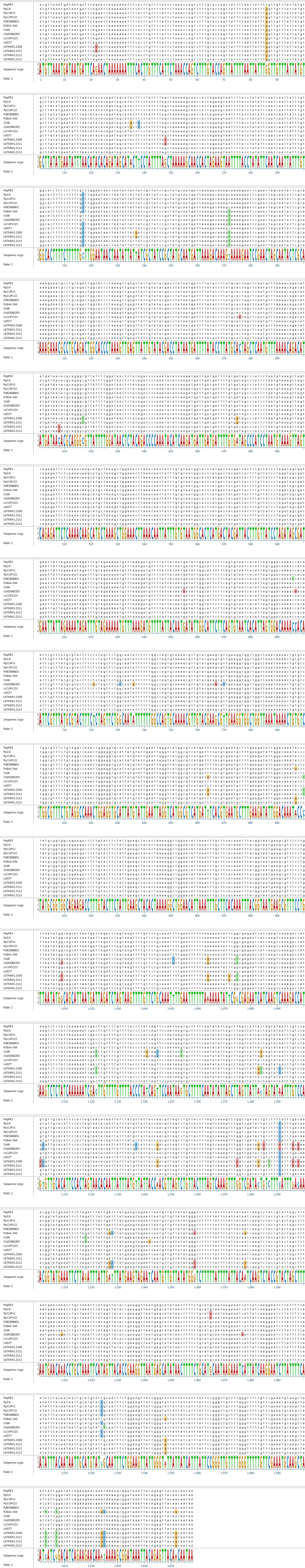
**

**
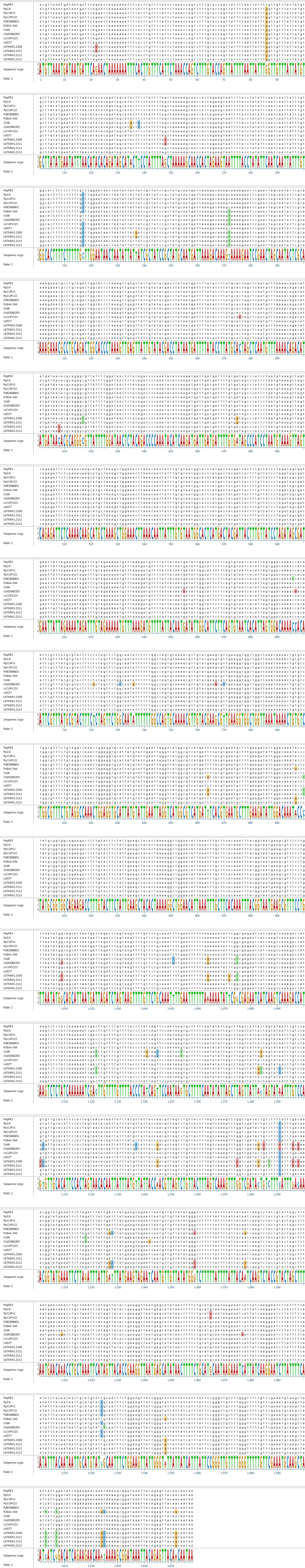
**

**Supplementary Figure 1.** Multiple alignment of the nucleotide sequences of the *gtf* genes from lactic acid bacteria. Colored residues are those differing from the equivalents in the reference *P. ethanolidurans* CUPV141 *gtf* gene.


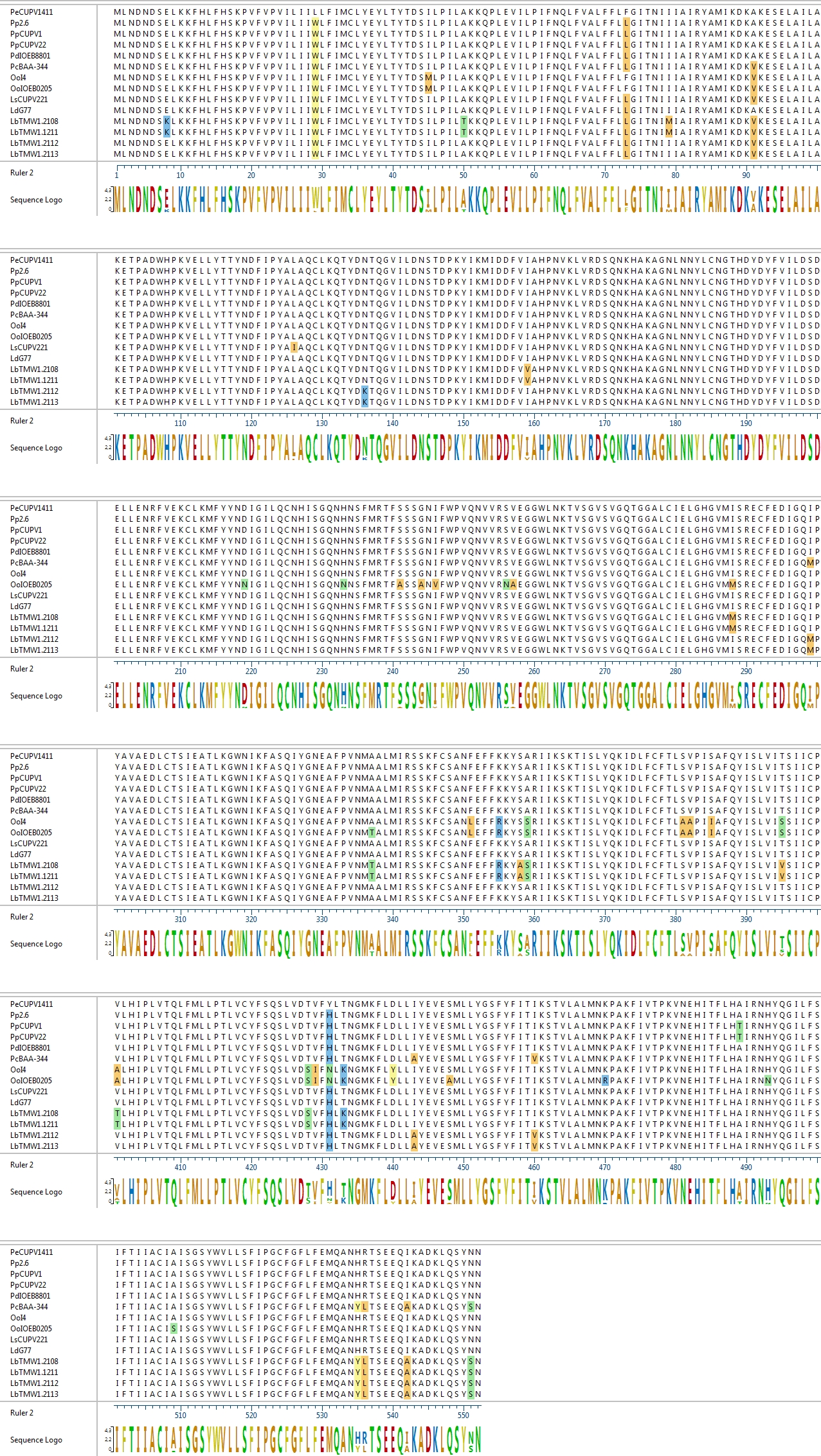


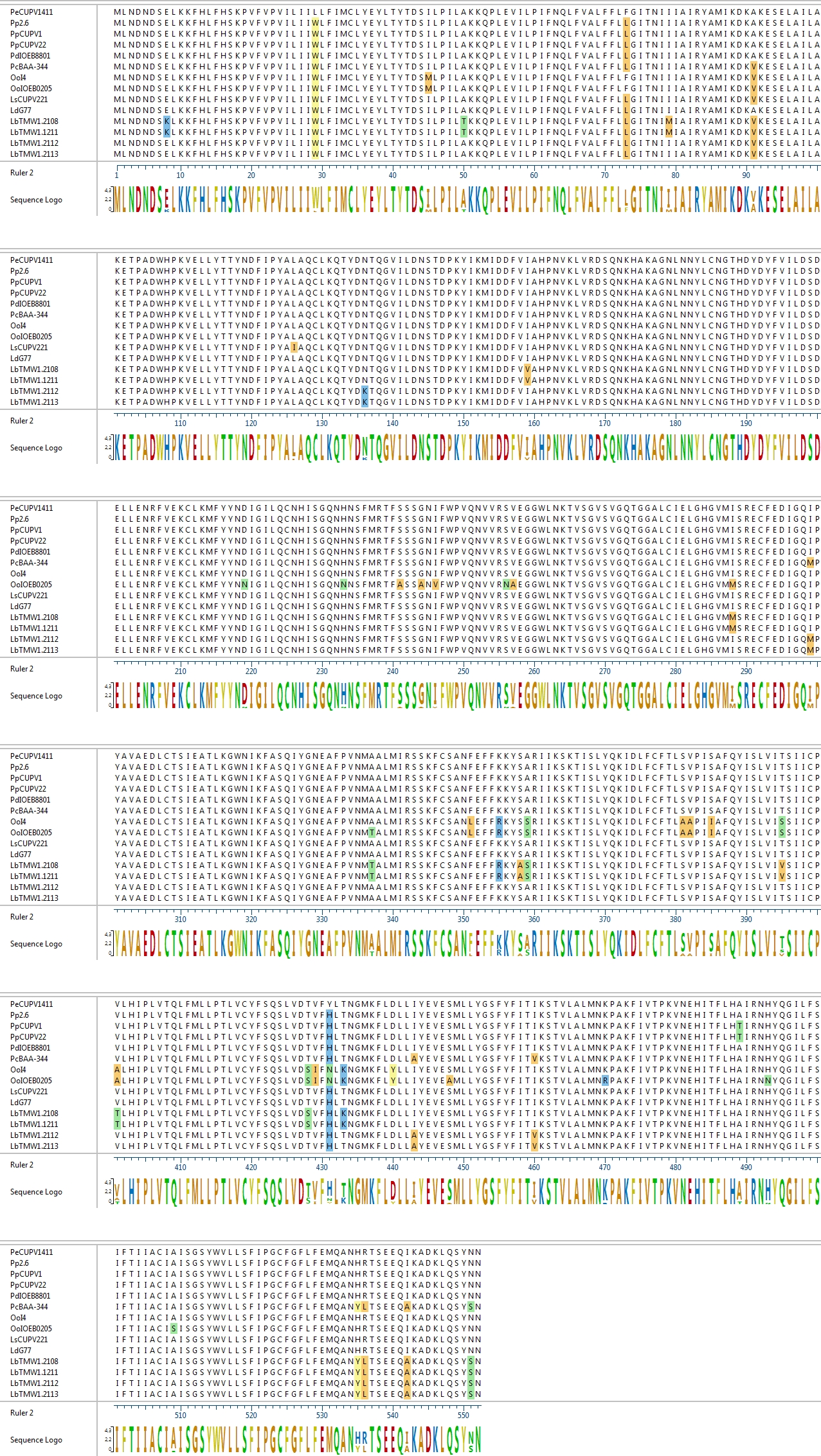


**Supplementary Figure 2.** Multiple alignment of the amino acids sequences of GTF glycosyltransferases from lactic acid bacteria. Colored residues are those differing from the equivalents in the reference *P. ethanolidurans* CUPV141 GTF enzyme.


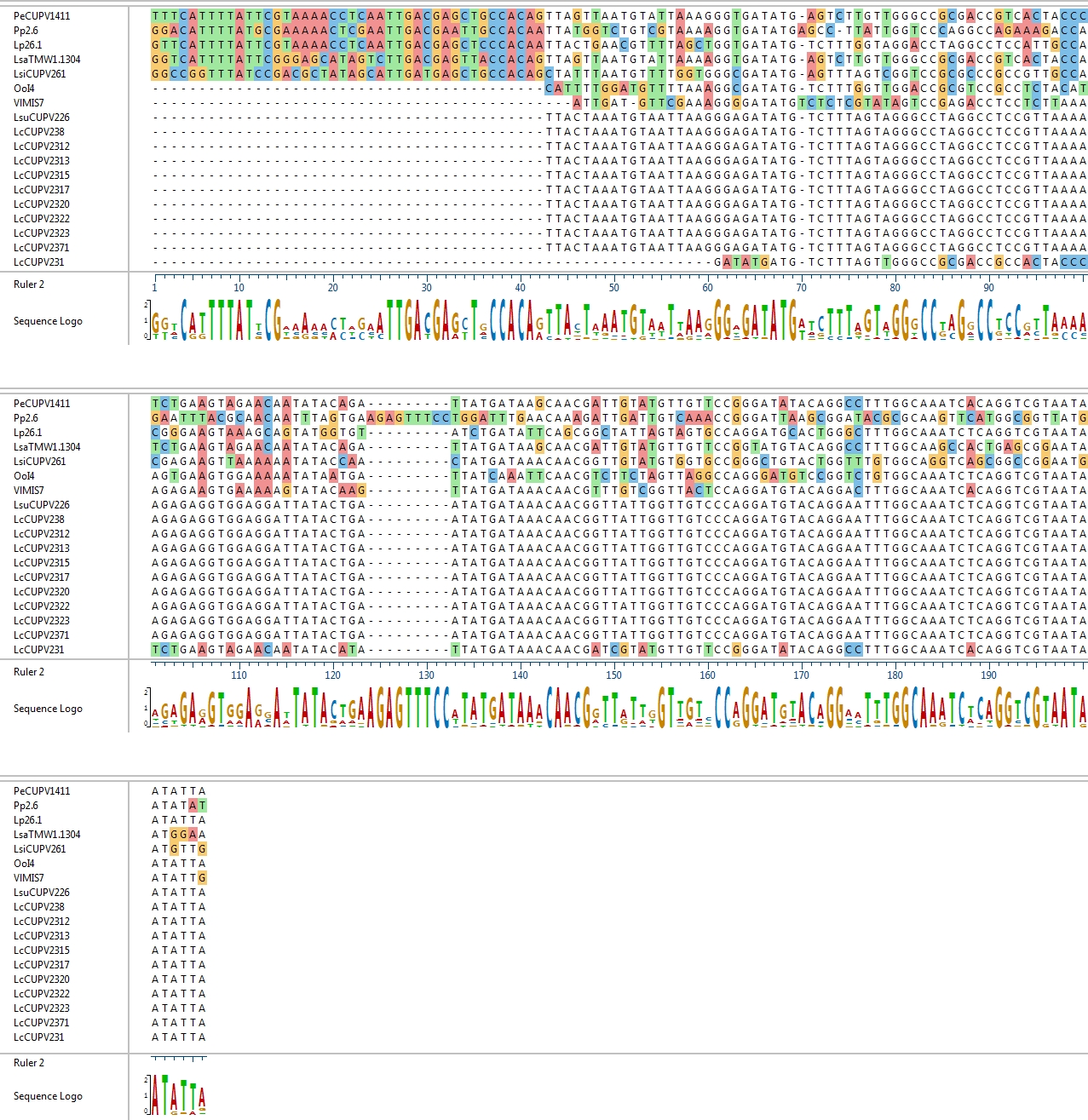


**Supplementary Figure 3.** Multiple alignment of the nucleotide sequences of the *p-gtf* gene from lactic acid bacteria. Colored residues are are those differing from the consensus nucleotidic sequence, which was generated with the nucleotides present in at least five *p-gtf* genes.


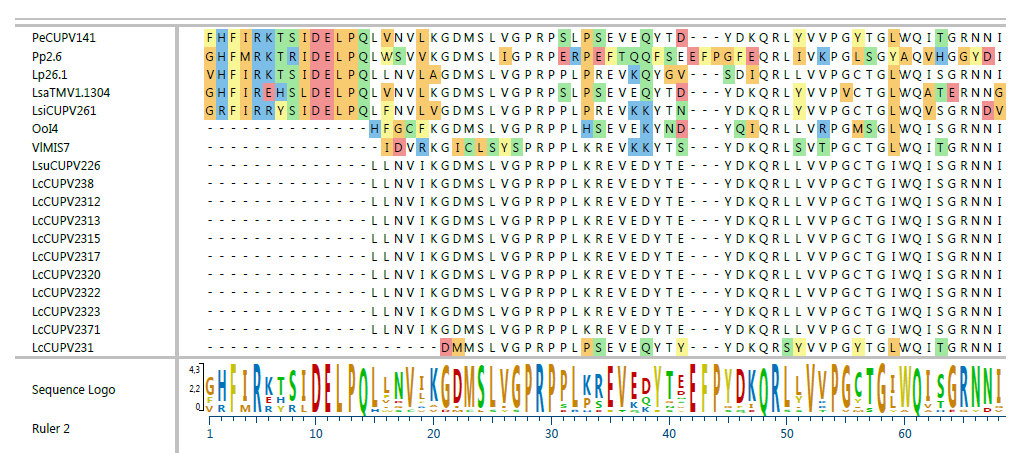


**B C**

**Supplementary Figure 4.** Multiple alignment of the amino acids sequences of the p*-*GTF from lactic acid bacteria. Colored residues are those differing from the consensus amino acid sequence, which was generated with the amino acids present in at least five polypeptides. Black arrows indicate the glutamate (E) and tyrosine (Y) residues, which could be involved in catalysis.


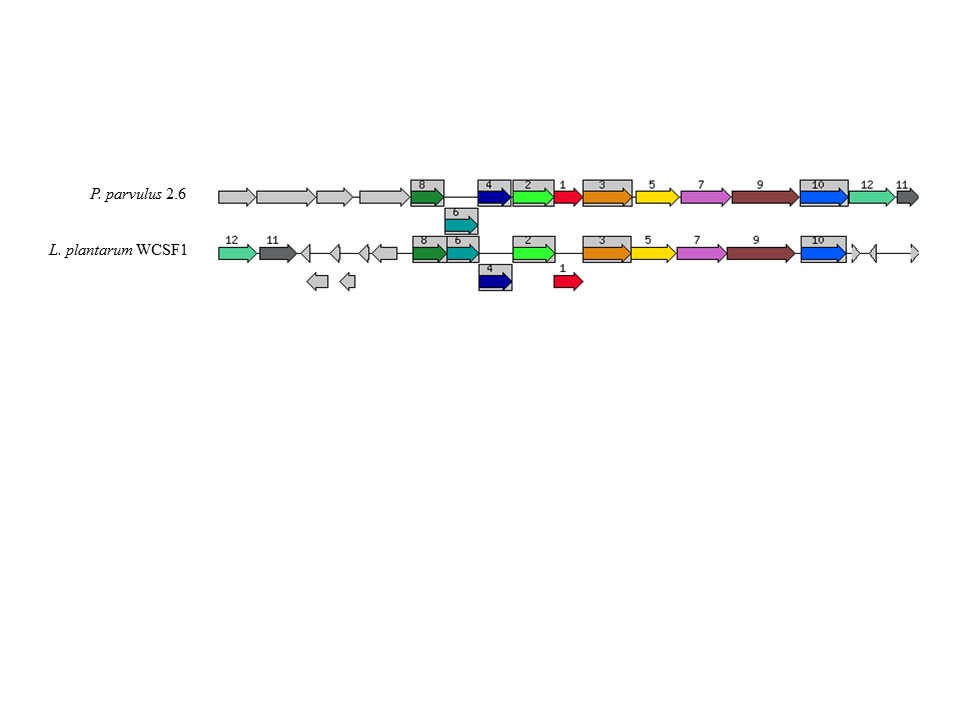


**Supplementary Figure 5.** Cluster (eleven genes) encoding the proteins responsible for the synthesis and secretion of the HePS of *P. parvulus* 2.6. A similar cluster is present in *L. plantarum* WCSF1. 1. Undecaprenyl-phosphate galactosephosphotransferase (EC 2.7.8.6); 2. UDP-glucose 4-epimerase (EC 5.1.3.2); 3. Exopolysaccharide biosynthesis glycosyltransferase EpsF (EC 2.4.1.-); 4. Manganese-dependent protein-tyrosine phosphatase (EC 3.1.3.48); 5. Glycosyltransferase; 6. Tyrosine-protein kinase EpsD (EC 2.7.10.2); 7. Polysaccharide polymerase; 8. Tyrosin-protein kinase transmembrane modulator EpsC; 9. O-antigen flippase Wzx; 10. Capsular polysaccharide biosynthesis protein; 11. dTDP-4-dehydrorhamnose reductase (EC 1.1.1.133); 12. d-TDP-glucose 4,6-dehydratase (EC 4.2.1.46).
